# Validation of an Automated Artificial Intelligence Algorithm for the Quantification of Major OCT Parameters in Diabetic Macular Edema

**DOI:** 10.3390/jcm12062134

**Published:** 2023-03-09

**Authors:** Edoardo Midena, Lisa Toto, Luisa Frizziero, Giuseppe Covello, Tommaso Torresin, Giulia Midena, Luca Danieli, Elisabetta Pilotto, Michele Figus, Cesare Mariotti, Marco Lupidi

**Affiliations:** 1 Department of Ophthalmology, University of Padova, 35128 Padova, Italy; 2 IRCCS—Fondazione Bietti, 00198 Rome, Italy; 3 Ophthalmology Clinic, Department of Medicine and Science of Ageing, University G. D’Annunzio Chieti-Pescara, 66100 Chieti, Italy; 4 Department of Surgical, Medical and Molecular Pathology and Critical Care Medicine, University of Pisa, 56126 Pisa, Italy; 5 Eye Clinic, Department of Experimental and Clinical Medicine, Polytechnic University of Marche, 60126 Ancona, Italy

**Keywords:** diabetic macular edema, artificial intelligence, biomarker, spectral domain OCT, outcomes, subretinal fluid, intraretinal fluid, hyperreflective retinal foci, external limiting membrane, ellipsoid zone

## Abstract

Artificial intelligence (AI) and deep learning (DL)-based systems have gained wide interest in macular disorders, including diabetic macular edema (DME). This paper aims to validate an AI algorithm for identifying and quantifying different major optical coherence tomography (OCT) biomarkers in DME eyes by comparing the algorithm to human expert manual examination. Intraretinal (IRF) and subretinal fluid (SRF) detection and volumes, external limiting-membrane (ELM) and ellipsoid zone (EZ) integrity, and hyperreflective retina foci (HRF) quantification were analyzed. Three-hundred three DME eyes were included. The mean central subfield thickness was 386.5 ± 130.2 µm. IRF was present in all eyes and confirmed by AI software. The agreement (kappa value) (95% confidence interval) for SRF presence and ELM and EZ interruption were 0.831 (0.738–0.924), 0.934 (0.886–0.982), and 0.936 (0.894–0.977), respectively. The accuracy of the automatic quantification of IRF, SRF, ELM, and EZ ranged between 94.7% and 95.7%, while accuracy of quality parameters ranged between 99.0% (OCT layer segmentation) and 100.0% (fovea centering). The Intraclass Correlation Coefficient between clinical and automated HRF count was excellent (0.97). This AI algorithm provides a reliable and reproducible assessment of the most relevant OCT biomarkers in DME. It may allow clinicians to routinely identify and quantify these parameters, offering an objective way of diagnosing and following DME eyes.

## 1. Introduction

Diabetic macular edema (DME) represents a major cause of vision loss among working-aged individuals in developed countries [1,2]. At present, approximately 537 million adults (20–79 years) are living with diabetes, and this number is projected to continuously rise [3]. The prevalence of DME among individuals with diabetes in Europe was estimated to be 3.7%, and its pooled mean annual incidence in type-2-diabetes patients was 0.4% [4]. DME is a multifactorial and complex disease driven by hypoxia, inflammation, hyperpermeability, and angiogenesis [5,6]. As a consequence, it is reasonable to hypothesize different DME phenotypes with different disease severity, risk of progression, and treatment outcomes [7]. Therefore, the assessment of the individual morphologic characteristics of DME may provide a better understanding of the pathophysiology of this disease, which, in turn, might help in the selection of the best treatment option in a personalized precision medicine approach [8].

Optical coherence tomography (OCT), particularly spectral domain OCT (SD-OCT), has entailed a significant improvement in the diagnostic efficacy of DME [9,10]. Qualitative and quantitative morphological features obtained via SD-OCT provide relevant information about changes at the level of the internal and external retina, identifying the biological limits of therapeutic interventions [11].

Among major OCT features, the presence and amount of intraretinal (IRF) and subretinal fluid (SRF), the integrity of the external limiting membrane (ELM) and ellipsoid zone (EZ), and the number of hyperreflective foci (HRF) have been identified as the most currently reliable OCT biomarkers for DME diagnosis and prognosis [12,13,14,15,16,17,18,19].

Over the last several years, significant advances in telecommunications, artificial intelligence (AI), and deep learning (DL)-based systems have opened new horizons for creating efficient tools for the quantification of major parameters in macular disorders [20,21,22]. The literature data suggest that AI may achieve high performance in detecting retinal fluid [23,24] and in assessing anatomic changes over the course of the disease [25]. Moreover, particularly in eyes with age-related macular degeneration (AMD), AI has demonstrated its capability to detect both qualitatively and quantitatively the presence of IRF and SRF in a real-world scenario [26].

This study aims to report the validation and applicability of an AI algorithm for the identification and quantification of the currently most significant OCT biomarkers in DME.

## 2. Materials and Methods

### 2.1. Study Design and Dataset

A multicenter AI algorithm validation study was performed in DME eyes. The study was conducted in accordance with the rules of the Declaration of Helsinki. Since all scans were completely anonymized, informed consent was waived by the Istitutional Review Board for the image analysis. SD-OCT scans of eyes affected by DME from type 1 and 2 patients were collected from four different Italian reference centers for diabetic retinopathy and maculopathy. All SD-OCT scans images were obtained using the Spectralis HRA + OCT2 platform (Heidelberg Engineering, Heidelberg, Germany). For each study eye, a volumetric map and a linear scan were analyzed. Inclusion criteria for OCT scans were as follows: volumetric scan of 49 scans in High-Speed (HS) mode >12 Automatic Real-Time Tracking (ART) (quality index > 28) and linear scan passing through the fovea acquired in High-Resolution (HR) mode >90 ART (quality index > 30) were analyzed. Exclusion criteria were any sign of chorioretinal diseases other than diabetic macular edema (e.g., drusen).

At any site, the scan of each eye was separately analyzed by the automatic quantification AI software and by clinical evaluation. 

### 2.2. AI Algorithm Description and Analysis

**Figure 1:**
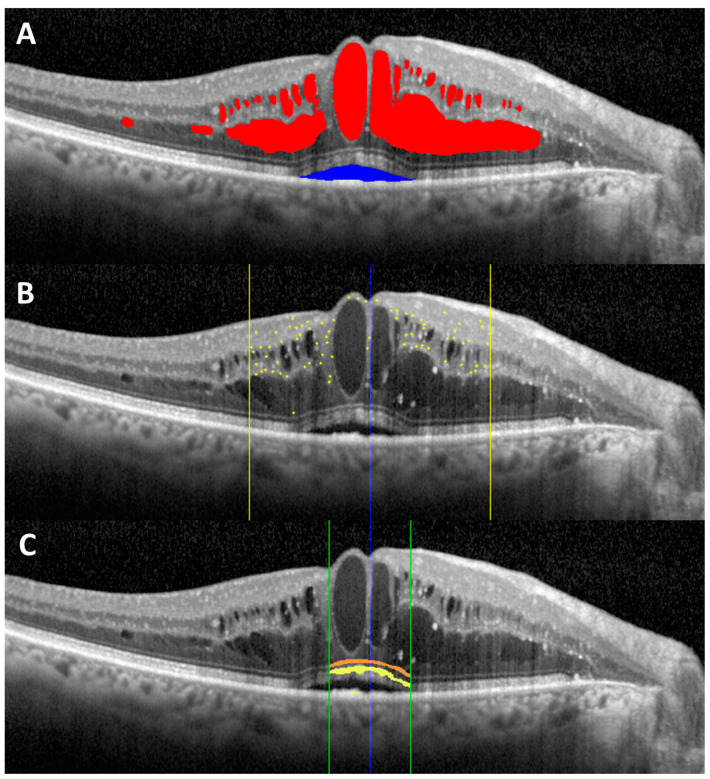
Overview of the different spectral domain optical coherence tomography biomarkers evaluated: (**A**) intraretinal fluid (red) and subretinal fluid (blue); (**B**) hyperreflective retinal foci (yellow dots) localized within the central 3 mm (yellow lines); (**C**) external limiting membrane (orange) and ellipsoid zone (yellow) localized within the central 1 mm (green lines).

The AI algorithm is based on adversarial generative networks, which is a DL technique that uses a small portion of labeled data (manually defined by the clinicians) and lots of unlabeled data to build a fully labeled dataset propagating the labels throughout the database. It is a semi-supervised learning AI capable of training itself on previously labeled datasets and predicting possible variations or noises that can characterize these datasets to perform effective diagnoses in a real-world context [27]. The AI algorithm is capable of evaluating separately different OCT biomarkers at the same time (Figure 1). The hardware/technical requirements for using the software have been summarized in Table 1. 

All OCT scans of each study eye were segmented using the AI automatic software. The data collected from the whole volumetric scan included IRF and SRF volumes; the percentage of IRF volume in the central 1 mm (IRF-1) circle, in the ring between 1 and 3 mm (IRF-3), and between 3 and 6 mm (IRF-6) was obtained. The percentage of external limiting membrane (ELM) and ellipsoid zone interruption (EZ) was analyzed in the central 1 mm of the central scan of the map, passing through the fovea. From the HR linear scan, the number of HRF (as previously described) in the central 3 mm was calculated [16].

### 2.3. Clinical Evaluation

**Figure 2:**
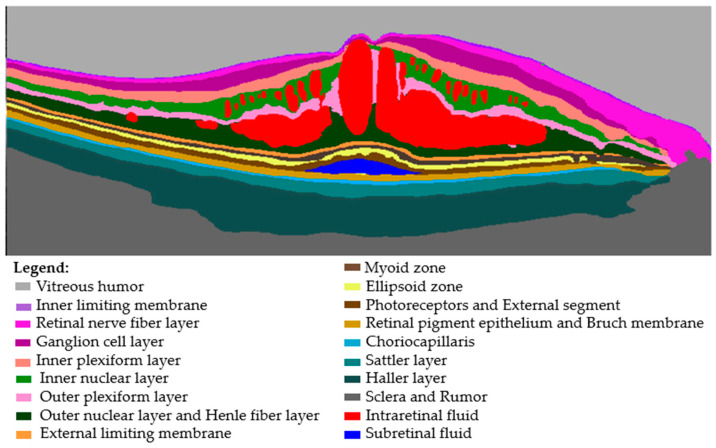
Retinal layer segmentation.

The clinical evaluation, which was performed by blinded, experienced examiners (TT, ML, LT, GC), assessed the presence of IRF, SRF, ELM, and/or EZ interruption (0 = absent, 1 = present). Moreover, for each of them, the quantification accuracy, according to the images, was evaluated as accurate or inaccurate. The number of HRF was manually counted (for all eyes) by one blinded, trained medical retinal expert, who worked in one reference center. Finally, for both volumetric and linear scans, quality parameters, namely the accuracy of the automated fovea centering and of the segmentation of retinal layers, were evaluated by a blinded medical retinal expert (accurate/non accurate) (Figure 2).

### 2.4. Outcomes

The primary outcome was the degree of agreement between the AI and the clinical evaluation for assessing the presence of IRF, SRF, ELM/EZ interruption, and the number of HRF. Secondary outcomes included the accuracy of the quantification of OCT biomarkers and quality OCT parameters.

### 2.5. Statistical Analysis

For all the analyses, SAS-STAT v.9.4 (SAS Institute, Cary, NC, USA) was used. 

The following parameters were considered in the present study: IRF, SRF, ELM, EZ, and HRF. Such parameters were summarized according to the usual indexes provided by descriptive statistics: mean and standard deviation for quantitative variables and absolute frequency and percentage for qualitative ones.

The validation process was conducted by comparing the assessment obtained by the AI system with the clinical evaluation. Moreover, the ROC curve of SRF versus clinical evaluation (gold standard) was calculated. The performance was expressed by means of the area under the curve (AROC). A SRF cutoff was identified considering the following: Youden index, Euclidean distance from 0,1, difference between sensitivity and specificity; and the total number of concordant evaluations (percentage over sample size). Kappa, PABAK, and AC1 indexes and their 95% confidence intervals were computed for the best cutoff. Agreement between AI system and clinical evaluations was calculated by means of kappa coefficient and two other indexes: PABAK, that is, prevalence- and bias-adjusted kappa coefficient, and Gwet’s AC1. Cohen’s kappa results are interpreted as follows: values ≤ 0 are interpreted as indicating no agreement, and values of 0.01–0.20 are interpreted as none to slight, 0.21–0.40 as fair, 0.41–0.60 as moderate, 0.61–0.80 as substantial, and 0.81–1.00 as almost perfect agreement [28].

HRF, which is a quantitative measure, was analyzed by means of the Bland–Altman graphic procedure. Intraclass Correlation Coefficient (ICC) and its 95% confidence interval were calculated as well [29].

## 3. Results

A total of 303 DME eyes were included in this analysis. Mean central subfield thickness was 386.5 ± 130.2 µm (range: 172–881 µm). By definition of macular edema, IRF was detected by the AI software in all eyes. The mean IRF volume, which was assessed by the AI software, was 0.898 ± 1.367 mm^3^ (range: 0.001–11.070 mm^3^): 0.069 ± 0.089 mm^3^ in the central 1 mm circle, 0.291 ± 0.422 mm^3^ in the 3 mm ring, and 0.538 ± 0.919 mm^3^ in the 6 mm ring. The distribution, in terms of percentage, of IRF was 13.9 ± 18.0% in the central circle, 34.4 ± 21.9% in the 3 mm ring, and 51.3 ± 30.2% in the 6 mm ring. IRF density (%/relative surface area) was 0.088 ± 0.114 in the central circle, 0.047 ± 0.068 in the 3 mm ring, and 0.025 ± 0.043 in the 6 mm ring.

Regarding SRF, the volume was computed as SRF if the likelihood of SRF presence was above a predetermined threshold (≥ 0.002 mm^3^, sensitivity: 89.7%, specificity: 97.0%; Youden index: 0.867). SRF was detected in 43 eyes by the software, and the mean volume was 0.111 ± 0.191 mm^3^ (range: 0.002–0.848 mm^3^). 

ELM and/or EZ interruption were detected by the AI software in 70 (23.1%) and 111 (36.6%) eyes, respectively. The mean percentage of interruption in the central 1 mm was 38.4 ± 30.4 and 41.4 ± 34.9 for ELM and EZ, respectively.

The mean number of HRF automatically counted by the AI software in the central 3 mm of the HR linear scan were 71.9 ± 22.8.

Table 2 shows the main OCT findings.

### 3.1. Agreement between AI Software and Clinical Evaluation 

The observed agreement between AI software and clinical evaluation ranged between 0.960 for SRF volume and 0.977 for ELM integrity. Kappa inter-rater agreement (95% confidence interval) was 0.831 (0.738–0.924) for SRF volume, 0.934 (0.886–0.982) for ELM integrity, and 0.936 (0.894–0.977) for EZ integrity (Table 3).

**Figure 3:**
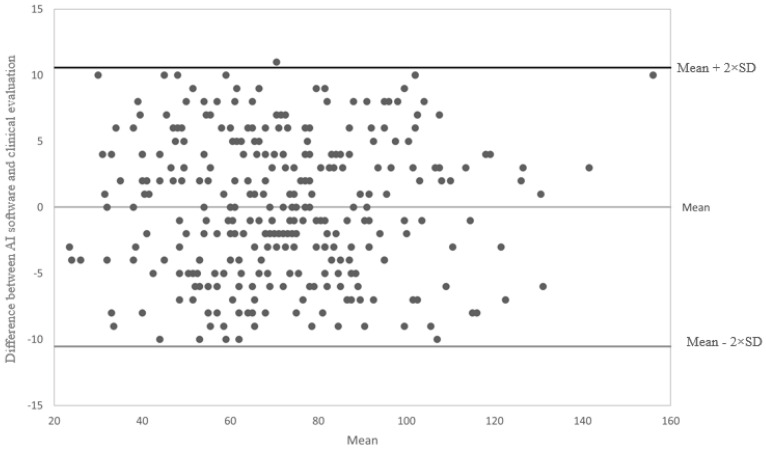
Bland–Altman plot analysis comparing the number of hyperreflective foci (HRF) assessed by artificial intelligence (AI) software and clinical evaluation. AI: artificial intelligence; SD: standard deviation.

Bland–Altman plot analysis was used to assess the agreement between AI software and clinical evaluation for HRF count (Figure 3). In the Bland–Altmann plot, almost all measured differences were in the range (±2 SD), with a mean difference between the clinical and automatic count of 0.03 ± 5.277. No significant trend was evident. The intraclass correlation coefficient was 0.973 (95% confidence interval: 0.966 to 0.979).

### 3.2. Quantification Accuracy

The automatic quantification was defined clinically accurate in 289 (95.38%) eyes for IRF and 287 (94.72%) for SRF. Regarding ELM and EZ, the rate of interruption was found clinically (qualitatively) accurate in 290 (95.71%) and 288 (95.05%) eyes for ELM and EZ, respectively. The degree of accuracy did not show any difference among the different study centers (Table 4).

### 3.3. Quality Parameters

In foveal identification, 1 eye in the map and no eye in the linear scan were assessed as inaccurate on all of the 303 eyes. The automatic retinal layer segmentation was identified as clinically inaccurate in 3 and 1 of 303 eyes in the map and the linear scan, respectively (Table 4). 

## 4. Discussion

As the global population ages, many relevant medical and social demographic problems emerge. It leads to an increase in population morbidity and mortality because the prevalence of chronic and degenerative diseases increases with age [30]. This entails that health systems must cope with increasing demand with limited resources, both human and material [31].

As previously mentioned, the prevalence of diabetes and diabetes-related complications has continued to increase globally [3]. The high prevalence of DME not only seriously affects people’s life quality but also lays a heavy economic burden on healthcare budgets [3,4].

These facts have generated a growing interest in the development of software-based analysis using AI. DL algorithms empower computers to suggest diagnosis or clinical management without direct human intervention by extracting clinically relevant information from medical data [31].

Therefore, there is a need to develop tools that carry out fast, accurate, reliable, safe, and cost-effective evaluations that allow for the optimization of health resources.

The current paper evaluated the effectiveness and reproducibility of new AI software for identifying and quantifying different SD-OCT biomarkers in DME. 

According to our results, there was an almost perfect agreement between the AI software and clinical evaluation for the SRF volume and ELM and EZ integrity. Additionally, the analysis comparing the number of HRF assessed by AI and clinical evaluation showed excellent reliability. The clinical relevance of these findings depends critically on the relationship between OCT biomarkers and clinical diagnosis and outcomes. OCT imaging has become the gold standard for the diagnosis and grading of DME. Current evidence shows that different OCT biomarkers (IRF, SRF, ELM/EZ integrity, and HRF) are particularly related to DME outcomes, even more than the central retinal thickness [12,13,14,15,16,17,18,19,25,26,32,33]. Although these OCT biomarkers have been correlated with both pre-treatment and post-treatment anatomic and functional outcomes (e.g., retinal thickness and visual acuity), they have different pathogenetic characteristics and, therefore, represent different aspects (phenotypes) of DME. Additionally, current unassisted identification and quantification of these OCT biomarkers, although clinically useful, are still subjective and manual forms of assessment, and in particular, quantification of imaging biomarkers becomes difficult to implement in daily clinical practice, making it unfeasible in the clinical setting, as shown in AMD [34]. Therefore, automatic image analysis is needed in order to provide objective and reproducible measurements of quantitative features.

AI has been successfully used for diabetic retinopathy screening, using fundus photos, even if with variable protocols, allowing early detection, with the subsequent reduction in blindness and cost savings [35,36]. 

AI previous algorithms have been considered to follow changes of IRF and SRF volumes over time, which may aid clinicians to assess disease activity and treatment response [25,26,33]. Although it is incredibly difficult to make comparisons between different algorithms used in the different studies, our results about an AI quantification approach have shown excellent accuracy and reproducibility of this AI algorithm in DME. Furthermore, this AI software simultaneously provides the quantification of the currently most recognized biomarkers in DME. A number of other OCT biomarkers have been proposed in the literature, e.g., the disorganization of inner retinal layers (DRIL); however, the biomarkers analyzed by the reported AI software were the ones with the most robust evidence in terms of physiopathological and clinical meaning and defined OCT characteristics for detection. Finally, our findings clearly confirmed a center and operator independence.

The main purpose of this study was to assess the effectiveness of this AI algorithm for quantifying OCT biomarkers in DME, but it has not yet been applied on follow-up data of the same eyes obtained from different time points. This subject will be addressed in the ongoing follow-up study in order to analyze the performance of monitoring modifications of biomarkers over time, with the possible onset, for example, of more significant atrophic changes, which may represent a challenging issue for clinical practice. Another limitation of the study is that it did not evaluate any relationship between OCT and functional outcomes or other morphologic parameters. In fact, the aim of the study was to investigate the performance of the AI software in comparison with clinical evaluation by analyzing the most recognized DME biomarkers in the currently most frequently used scans (map and linear) in clinical practice.

## 5. Conclusions

The results of this study suggest that the proposed AI algorithm is a reliable and reproducible tool for detecting and quantifying different OCT biomarkers in DME eyes, which is currently considered prognostic even for the treatment outcomes.

Artificial intelligence may facilitate the quantification of these biomarkers in daily practice since it has been shown to be as accurate and precise as clinical evaluation but less time-consuming. Further studies are needed to implement this AI software in large real-world settings to assess changes over time and the clinical relationship between those changes and the course of the disease.

## Figures and Tables

**Table 1:** Overview of the hardware/technical requirements for using the AI software.

Hardware/Technical Features	Requirements
Physical memory space available in the computer	At least 4 GB
Operating system	Windows 10 or higher 64-bit
RAM memory in the computer	8 GB or more
Input images	Horizontal B scans and/or volumetric scans composed of parallel horizontal B scans
Linear scans (High Resolution mode)	ART > 90, Quality Index > 30
Volumetric scans	ART > 12, Quality Index > 28

GB: gigabytes; RAM: random access memory; ART: automatic real-time tracking.

**Table 2:** Overview of the main spectral domain optical coherence tomography (SD-OCT) features.

	AI algorithm	Clinicians	*p* Value ^a^
IRF, mm^3^ Mean ± SD Range	8.98 ± 13.67 0.00–110.7	N.A.	N.A.
SRF *, n (%) Absent Present	260 (85.8) 43 (14.2)	264 (87.1) 39 (12.9)	0.9811
ELM interruption, n (%) Absent Present	233 (76.9) 70 (23.1)	236 (77.9) 67 (22.1)	0.1660
EZ interruption, n (%) Absent Present	192 (66.4) 111 (36.6)	195 (64.4) 108 (35.6)	0.1646
HRF, n Mean ± SD Range	71.9 ± 22.8 22.0–161.0	71.9 ± 22.7 25.0–151.0	1.0000 ^b^

^a^ Fisher exact test. ^b^ Independent sample Student *t*-test. * Cut-off value: 0.02 mm^3^. AI: artificial intelligence; IRF: intraretinal fluid; SRF: subretinal fluid; ELM: external limiting membrane; EZ: ellipsoid zone; HRF: hyperreflective foci; SD: standard deviation; n: number; N.A.: not applicable.

**Table 3:** Inter-rater agreement κ between artificial intelligence (AI) software and clinical evaluation.

Parameter	+ +	+ −	− +	− −	p_0_	BI	PI	Kappa	PABAK	AC_1_
SRF	35	8	4	256	0.96	0.01	0.73	0.83 (0.74–0.92)	0.92 (0.88–0.97)	0.95 (0.92–0.98)
ELM	65	5	2	231	0.98	0.01	0.55	0.93 (0.89–0.98)	0.95 (0.92–0.99)	0.96 (0.94–0.99)
EZ	105	6	3	189	0.97	0.01	0.28	0.94 (0.89–0.98)	0.94 (0.90–0.98)	0.95 (0.91–0.98)

++ = classified “Present” by both modalities; *− −* = classified “Absent” by both modalities; + *−* and *−* + = discordant classifications: classified “Present” by AI software and “Absent” by clinical evaluation, and classified “Absent,” by AI software and “Present” by clinical evaluation, respectively; p_0_ = observed agreement; BI = bias index; PI = prevalence index; PABAK = prevalence-adjusted and bias-adjusted kappa; AC_1_ = Gwet’s first-order agreement coefficient. SRF: subretinal fluid; ELM: external limiting membrane; EZ: ellipsoid zone.

**Table 4:** Biomarker quantification accuracy and quality parameters in the overall study sample and among the different study centers.

	SD-OCT Measurements					
Biomarker, n (%)	Among Centers				*p* ^a^	Overall
	Center 1	Center 2	Center 3	Center 4		
IRF Accurate Not-accurate	71 (94.7) 4 (5.3)	77 (98.7) 1 (1.3)	71 (94.7) 4 (5.3)	70 (93.3) 5 (6.7)	0.3543	289 (95.4) 14 (4.6)
SRF Accurate Not-accurate	71 (94.7) 4 (5.3)	74 (94.9) 4 (5.1)	68 (90.7) 7 (9.3)	74 (98.7) 1 (1.3)	0.1808	287 (94.7) 16 (5.3)
ELM Accurate Not-accurate	72 (96.0) 3 (4.0)	74 (94.9) 4 (5.19	71 (94.7) 4 (5.3)	73 (97.3) 2 (2.7)	0.9241	290 (95.7) 13 (84.3)
EZ Accurate Not-accurate	72 (96.0) 3 (4.0)	74 (94.9) 4 (5.1)	71 (94.7) 4 (5.3)	71 (94.7) 4 (5.3)	1.0000	288 (95.1) 15 (4.9)
	**SD-OCT Quality Parameters**					
	**Among Centers**				***p* ^a^**	**Overall**
	**Center 1**	**Center 2**	**Center 3**	**Center 4**		
FCM Accurate Not-accurate	75 (100.0) 0 (0.0)	78 (100.0) 0 (0.0)	74 (98.7) 1 (1.39)	75 (100.0) 0 (0.0)	0.7426	302 (99.7) 1 (0.3)
FCL Accurate Not-accurate	75 (100.0) 0 (0.0)	78 (100.0) 0 (0.0)	75 (100.0) 0 (0.0)	75 (100.0) 0 (0.0)	1.0000	303 (100.0) 0 (0.0)
LSM Accurate Not-accurate	72 (96.0) 3 (4.0)	78 (100.0) 0 (0.0)	75 (100.0) 0 (0.0)	75. (100.0) 0 (0.0)	0.0441	300 (99.0) 3 (1.0)
LSL Accurate Not-accurate	74 (98.7) 1 (1.39)	78 (100.0) 0 (0.0)	75 (100.0) 0 (0.0)	75 (100.0) 0 (0.0)	0.7426	302 (99.7) 1 (0.3)

^a^ Fisher exact test. SD-OCT: spectral domain optical coherence tomography; IRF: intraretinal fluid; SRF: subretinal fluid; ELM: external limiting membrane; EZ: ellipsoid zone; FCM: fovea centering at map; FCL: fovea centering at linear scan: LSM: layer segmentation at map; LSL: layer segmentation at linear scan; n: number.

## Data Availability

The data presented in this study are available on reasonable request from the corresponding author.
